# Beneficial and Harmful Interactions of Antibiotics with Microbial Pathogens and the Host Innate Immune System

**DOI:** 10.3390/ph3051694

**Published:** 2010-05-25

**Authors:** Ronald Anderson, Gregory Tintinger, Riana Cockeran, Moliehi Potjo, Charles Feldman

**Affiliations:** 1 Medical Research Council Unit for Inflammation and Immunity, Department of Immunology, Faculty of Health Sciences, University of Pretoria and Tshwane Academic Division of the National Health Laboratory Service, Pretoria, South Africa; E-Mails: grtintinger@gmail.com (G.T.); riana.cockeran@up.ac.za (R.C.); moliehi.potjo@nhls.ac.za (M.P.); 2 Division of Pulmonology, Department of Internal Medicine, Steve Biko Pretoria Academic Hospital and Faculty of Health Sciences, University of Pretoria, Pretoria, South Africa; 3 Division of Pulmonology, Department of Internal Medicine, Charlotte Maxeke Johannesburg Academic Hospital and Faculty of Health Sciences, University of the Witwatersrand, Johannesburg, South Africa; E-Mail: feldmanc@medicine.wits.ac.za (C.F.)

**Keywords:** anti-inflammatory, fluoroquinolones, imidazole anti-mycotics, macrolides, mucociliary escalator, pattern recognition receptors, pro-inflammatory, tetracyclines

## Abstract

In general antibiotics interact cooperatively with host defences, weakening and decreasing the virulence of microbial pathogens, thereby increasing vulnerability to phagocytosis and eradication by the intrinsic antimicrobial systems of the host. Antibiotics, however, also interact with host defences by several other mechanisms, some harmful, others beneficial. Harmful activities include exacerbation of potentially damaging inflammatory responses, a property of cell-wall targeted agents, which promotes the release of pro-inflammatory microbial cytotoxins and cell-wall components. On the other hand, inhibitors of bacterial protein synthesis, especially macrolides, possess beneficial anti-inflammatory/cytoprotective activities, which result from interference with the production of microbial virulence factors/cytotoxins. In addition to these pathogen-directed, anti-inflammatory activities, some classes of antimicrobial agent possess secondary anti-inflammatory properties, unrelated to their conventional antimicrobial activities, which target cells of the innate immune system, particularly neutrophils. This is a relatively uncommon, potentially beneficial property of antibiotics, which has been described for macrolides, imidazole anti-mycotics, fluoroquinolones, and tetracyclines. Although of largely unproven significance in the clinical setting, increasing awareness of the pro-inflammatory and anti-inflammatory properties of antibiotics may contribute to a more discerning and effective use of these agents.

## 1. Introduction

In their review published in 1981, Miller and North stated that “immunocompetent individuals were the major beneficiaries of advances in antimicrobial chemotherapy” [1]. In the intervening thirty years, compelling evidence has emerged, largely as a result of the often limited efficacy of antibiotics in patients with primary and acquired immunodeficiency disorders, that eradication of microbial pathogens is indeed critically dependent on cooperative interactions between antimicrobial agents and host defences. In this setting, antibiotics weaken and disable microbial pathogens, enabling elimination by host defences. This is a common characteristic of antibiotics. Antibiotics, however, also interact with host defences in other ways, some potentially harmful, others beneficial. In the case of the former, some classes of antimicrobial agent may exacerbate inflammatory responses, leading to tissue damage and organ dysfunction in severe infection, while others possess anti-inflammatory activities, which may attenuate over-exuberant inflammatory reactions. These pro- and anti-inflammatory properties of antibiotics merit serious consideration when antimicrobial agents are selected for empiric therapy in patients with serious infections such as sepsis and community-acquired pneumonia, in whom outcome is frequently poor despite seemingly appropriate antimicrobial chemotherapy.

In this review, our primary objective is to evaluate the mechanisms, both pro-inflammatory and anti-inflammatory/cytoprotective, by which antibiotics interact with innate host defences, specifically the mucociliary escalator and those involving pattern recognition receptors and mobilization and activation of circulating neutrophils. This is preceded by a brief consideration of innate host defence mechanisms.

## 2. Innate Immune Mechanisms

These are the first-line host defences encountered by potentially invasive microbial pathogens. Notwithstanding the mechanical, physical and chemical components shown in Table 1, the major contributors to innate immunity are the mucociliary escalator and inflammatory reactions triggered via recognition of shared, conserved microbial molecules by pattern recognition receptors on epithelial cells and cells of the innate immune system.

**Table 1:** Mechanical, physical, and chemical components of the innate immune system.

Mechanical barriers presented by the skin and mucus membranes. Expulsive actions of coughing and sneezing, as well as peristalsis in the gastrointestinal tract. Washing effects of the body fluids, such as saliva, tears and urine. Unfavourable, acidic pH of the skin and stomach. Antimicrobial agents in body fluids such as lysozyme in tears and saliva, defensins in the skin and respiratory and gastrointestinal tracts, and surfactant proteins A and D in the lung. Elevated body temperatures, unfavourable for the growth of fastidious microbial pathogens.

### 2.1. Mucociliary escalator

While the contributions of cells such as neutrophils, macrophages, dendritic cells, mast cells and natural killer cells to innate immunity are well recognized, the important role of epithelial cells has been under-estimated [2,3]. The airway of the lung is continuously exposed to foreign particles, including inhaled microorganisms. Consequently, airway epithelial cells play a key role in innate defences, firstly by providing a mechanical barrier to inhaled microorganisms, secondly by signaling to cellular elements of the host defence system, and, thirdly, by direct killing of pathogens [2,3]. Both the upper and lower respiratory tracts are lined by a highly specialised ciliated columnar epithelium [2]. The cilia, together with the mucus layer above, constitute the mucociliary escalator that normally serves to keep the lower respiratory tract sterile. 

The primary function of the mucociliary escalator of the respiratory tract is therefore to prevent adhesion of microbial pathogens to respiratory epithelium, preventing colonization and subsequent progression to serious infection, either by translocation to the lower airways, or transcytosis across the epithelial barrier, enabling access to the bloodstream. Interference with adhesion to respiratory epithelium involves trapping of bacteria by mucopolysaccharides present in mucus released by the goblet cells of the airways. The mucus containing the entrapped bacteria is then moved upwards and outwards by the coordinated beating action of ciliated respiratory epithelium.

Colonization of the airway by microorganisms is an important precursor to the establishment of an infection. In order to evade host defences, however, bacteria entering the airway must adhere to the epithelium or epithelial-related structures [2]. Several studies have been undertaken to investigate the interaction of respiratory pathogens and their virulence factors with airway epithelium [2]. A number of bacterial pathogens that colonize the airways and cause respiratory tract infections, such as *Streptococcus pneumoniae*, *Haemophilus influenzae*, *Staphylococcus aureus*, *Klebsiella pneumoniae* and *Pseudomonas aeruginosa*, among many others, have been shown to produce factors which affect the ciliated epithelium [4,5,6,7,8,9,10,11,12,13]. These virulence factors appear to play a role in the pathogenesis of respiratory tract infections by disturbing ciliary structure or function, by stimulating mucus production and by damaging the structural integrity of the epithelium, thus enhancing colonization [5]. The consequence of this attenuated function may be persistent airway colonization, chronic infection or inflammation, or even bacterial proliferation, spread and invasion [14].

Host-derived factors that may cause ciliary dysfunction and epithelial injury during microbial infection include reactive oxygen species, in particular hydrogen peroxide, the endogenous bioactive phospholipids, platelet-activating factor (PAF), lyso-PAF and lysophosphatidylcholine (LPC), proteolytic enzymes, cytokines, leukotrienes, prostaglandins and many others [14].

### 2.2. Pattern recognition receptors

Pattern recognition receptors are the sentinels of the innate immune system. They are able to distinguish between “self” and “non-self” through recognition of highly conserved microbial structures such as lipopolysaccharides, lipoteichoic acids, and proteoglycans which are broadly presented on/in microbial pathogens, but not eukaryotic cells. Two of the most important categories of pattern recognition receptors are the Toll-like receptors (TLRs) and nucleotide oligomerization (NOD)-like receptors, which are present on/in epithelial cells and cells of the innate immune system [15,16]. Interaction of TLRs/NOD-like receptors with microbial pathogens triggers a cascade of events characterized by the production of pro-inflammatory cytokines/chemokines, release of histamine from tissue mast cells, localized up-regulation of the expression of adhesion molecules on vascular endothelium, complement activation via the alternative and mannan-binding lectin pathways, and resultant mobilization and influx of neutrophils. If it occurs promptly and efficiently, this early inflammatory response may either eradicate or contain the pathogen until adaptive host defences are mobilized. However, if mobilization of neutrophils occurs slowly and ineffectively, serious infection may ensue. 

## 3. Antibiotics and Inflammatory Responses

As mentioned above, some antibiotics exacerbate inflammatory responses, which is a potentially harmful activity, while others possess beneficial anti-inflammatory properties. In the case of the former group, these are generally cell-wall-targeted, bactericidal agents which cause the release of pro-inflammatory cell wall components and intracellular toxins normally released following bacteriolysis. Examples of these are lipopolysaccharides, lipoteichoic acids and pneumolysin, which exacerbate inflammatory reactions via their interactions with TLRs and NOD-like receptors on/in immune and inflammatory cells, activation of complement, and other mechanisms [17,18,19].

Antibiotic-mediated anti-inflammatory activity is achieved directly via antimicrobial activity and, in a few cases, by secondary anti-inflammatory properties which target immune and inflammatory cells. With respect to the former, antibiotics which inhibit bacterial protein synthesis, especially macrolides and macrolide-like agents, prevent the release of pro-inflammatory protein toxins from both Gram-positive and Gram-negative bacteria, as well as the production of other virulence factors, including bacterial adhesins and biofilm. Secondly, some classes of antibiotic, specifically macrolides, fluoroquinolones, imidazole anti-mycotics and tetracyclines, act directly on cells of the host defence system to inhibit the excessive production of several potent mediators of inflammation.

### 3.1. Pro-inflammatory and anti-inflammatory interactions of antibiotics with bacteria

As mentioned above, cell-wall active, bactericidal antibiotics, specifically beta-lactams, potentiate host-directed inflammatory responses against invasive bacterial pathogens via the release of pro-inflammatory intracellular toxins and cell-wall components from disintegrating bacteria. Such over-exuberant, poorly-controlled inflammatory responses pose the potential risk of inflammation-mediated damage to host cells and tissues. The harmful, pro-inflammatory activity of these antimicrobial agents has been demonstrated in many studies, either by measuring the release of intracellular toxins following exposure of bacteria to bactericidal agents *in vitro*, or in animal models of experimental infection which correlate survival with the anti-bacterial and pro-inflammatory potencies of antibiotics. For example, Hirst *et al.* reported that exposure of *Streptococcus pneumoniae* to penicillin *in vitro* resulted in bacteriolysis and exaggerated release of pneumolysin, which, as alluded to above, is a potent pro-inflammatory and cytotoxic virulence factor of the pneumococcus [20]. Pneumolysin both initiates and exacerbates harmful inflammatory reactions via its pore-forming actions on immune and inflammatory cells, its interaction with TLR4 on these cells, as well as by its complement-activating properties [17]. 

Much of our own work has focused on the interaction of macrolides (erythromycin, clarithromycin) and macrolide-like agents (azithromycin clindamycin, telithromycin) with macrolide-susceptible and macrolide-resistant strains of the pneumococcus. We have observed that these agents, at sub-MIC concentrations, are potent inhibitors of pneumolysin production by both susceptible and resistant strains of this microbial pathogen, with doxycycline being somewhat less effective, while amoxicillin, ceftriaxone, and tobramycin were ineffective [21,22]. Using a rabbit model of experimental pneumococcal meningitis, others have reported that administration of inhibitors of bacterial protein synthesis, but not beta-lactams, results in significant reductions in pneumolysin in the cerebrospinal fluid, an attenuated inflammatory response, and protection against neuronal injury [23,24,25]. Moreover, in a murine model of secondary, influenza-associated pneumococcal pneumonia, the lowest survival rate in antibiotic-treated animals was observed in those treated with ampicillin only, while the highest rates were noted in those treated with inhibitors of protein synthesis (azithromycin and clindamycin) only, or in combination with ampicillin [26]. Improved survival in the azithromycin/clindamycin-treated groups was associated with an attenuated inflammatory response, manifested as lower numbers of inflammatory cells and pro-inflammatory cytokines in the lungs, and less severe histopathological changes [26].

In addition to the aforementioned pro-inflammatory mechanisms, beta-lactam antibiotics at sub-MIC and MIC concentrations, have been reported to increase the production of the extracellular protein toxins, toxic-shock syndrome toxin-1 (TSST-1), Panton-Valentine leukocidin, and alpha-hemolysin by *Staphylococcus aureus* [27,28]. Somewhat paradoxically, given the bactericidal action of beta-lactams, augmentation of toxin production by *S. aureus* was found to result from increased transcription of genes encoding these proteins, possibly as an antibiotic-induced stress response [28]. In contrast, the inhibitors of protein synthesis, clindamycin and linezolid, were found to significantly attenuate toxin production by this microbial pathogen.

Beta-lactams, as well as other classes of bactericidal antibiotics, including fluoroquinolones, have also been reported to increase the release of shiga-like toxins from entero-haemorrhagic *Escherichia coli* (EHEH), predisposing patients for the development of serious complications such as haemolytic-uremic syndrome [29,30,31]. Pretreatment of EHEH with macrolides or clindamycin *in vitro* has been reported to attenuate the stimulatory effects of bactericidal antimicrobial agents on the release of shiga-like toxins [32].

Clearly, antibiotic selection based solely on the grounds of antimicrobial potency may be inappropriate in some clinical settings, particularly serious infections caused by toxin-producing pathogens with high bacterial loads. In this situation, circumstances permitting, administration of an inhibitor of bacterial protein synthesis, either prior to, or together with a compatible bactericidal agent may be justified to reduce the potential risk of an antibiotic-associated/potentiated inflammatory reaction [25]. Acceptance of such a strategy will, however, be dependent on the acquisition of compelling supportive evidence from multi-centre clinical trials. Nonetheless, interesting and potentially relevant precedents do exist. In a recent study, Giamarellos-Bourboulis and colleagues reported that addition of clarithromycin to standard Gram-negative antimicrobial therapy in patients with sepsis and ventilator-associated pneumonia (VAP), due overwhelmingly to Gram-negative pathogens, accelerated the resolution of VAP and weaning from mechanical ventilation in surviving patients, while delaying death in those who died of sepsis [33]. These authors proposed that the beneficial effects of adjunctive clarithromycin were due to the secondary anti-inflammatory activities of this agent described below. An additional strategy, for which good supportive evidence exists, is the use of corticosteroids as an adjunct to beta-lactams in the treatment of severe pneumococcal meningitis [34]. In these afore-mentioned settings, the primary activity of either macrolides or corticosteroids is to ameliorate the potentially hazardous inflammatory responses initiated by the causative microbial pathogens, which may, in turn, be exacerbated by the administration of bactericidal agents.

Aside from the pro-inflammatory mechanisms mentioned above, several other mechanisms have been described by which indiscriminate administration of antibiotics may have the opposite to intended effects. Firstly, in a murine model of experimental infection, it has been reported that broad-spectrum antibiotic-mediated elimination of commensal bacteria in the gastrointestinal tract resulted in down-regulation of the secreted C-type lectin RegIIIγ. This, in turn, led to increased susceptibility to infection with vancomycin-resistant enterococci [35]. Secondly, commensal microflora in the skin, especially staphylococcal species, down-regulate the production of pro-inflammatory cytokines by keratinoytes [36]. This is achieved by a mechanism involving release of lipoteichoic acid from the bacteria, which, via its interactions with TLR2, suppresses TLR3-mediated activation of these cells. The authors propose that elimination of the skin microflora by broad-spectrum antibiotics may lead to inflammatory complications of wound healing and inflammatory skin disorders [36].

## 4. Cytoprotection Due to Interaction of Antibiotics with Bacteria

Antibiotics, especially the macrolide group of antimicrobial agents, have been shown to attenuate the harmful effects of respiratory pathogens on mucociliary clearance and structural integrity of the epithelium. These beneficial, cytoprotective effects of antibiotics are achieved by two mechanisms: (i) antagonism of the adhesion of bacteria to epithelial cells; and (ii) inhibition of the production of bacterial cytotoxins, such as pneumolysin, which cause ciliary slowing and epithelial disruption (reviewed in [14]).

## 5. Anti-Inflammatory Interactions of Antibiotics with Innate Host Defences

Notwithstanding beneficial anti-inflammatory and cytoprotective activities of antibiotics consequent to the direct actions of these agents on their target pathogens, some classes of antimicrobial agent possess secondary anti-inflammatory properties, which are independent of their primary antimicrobial activities. Four classes of antibiotics, *viz.* macrolides, imidazole anti-mycotics, fluoroquinolones, and tetracyclines appear to possess secondary, anti-inflammatory properties which complement their primary antimicrobial activities. Of these, macrolides, imidazole anti-mycotics and tetracycline modulate the pro-inflammatory activities of neutrophils, primarily by targeting the production of neutrophil chemoattractants, while fluoroquinolones inhibit the production of pro-inflammatory cytokines.

### 5.1. Anti-inflammatory activities of macrolides

Macrolides have an extremely high level of tissue penetration. They are highly concentrated by epithelial cells and cells of the innate immune system and attenuate the pro-inflammatory activities of these cells, especially neutrophils. Macrolides inhibit the generation of reactive oxygen species by neutrophils and monocytes/macrophages, apparently by a membrane-stabilizing mechanism, targeting both NADPH oxidase and nitric oxide synthase (reviewed in [14]). They also possess anti-elastolytic activity, and promote apoptosis of neutrophils, thereby accelerating resolution of inflammation, while the expression of neutrophil adhesion molecules and their counter-receptors on vascular endothelium is also inhibited by these agents [14]. Macrolides are also effective inhibitors of production of the potent neutrophil chemoattractant, IL-8, by a variety of cell types, including bronchial epithelial cells, eosinophils, monocytes, fibroblasts, and airway smooth muscle cells (reviewed in [14]), which is thought to be the predominant anti-inflammatory activity of these agents [37]. Macrolides, specifically azithromycin, have also been reported to inhibit the synthesis of the neutrophil-activating cytokine, tumour necrosis factor (TNF), by airway epithelial cells [38]. Macrolide-mediated inhibition of synthesis of IL-8 and TNF appears to involve interference with intracellular signaling mechanisms which converge on transcriptional activation of gene expression. These mechanisms include inhibition of mitogen-activated protein kinases and extracellular signal-regulated kinases 1 and 2, as well as nuclear translocation of the transcription factors nuclear factor-kappa B and activator protein-1 [37,38,39,40,41]. 

Notwithstanding attenuation of the production of cytotoxic reactive oxygen species and proteases by activated phagocytes in the airways, macrolides also protect ciliated respiratory epithelium against the membrane-damaging actions of bioactive phospholipids such as PAF, lyso-PAF, and LPC released by activated neutrophils and other inflammatory cells. These cytoprotective effects of the macrolides are consequent to their membrane-stabilizing properties [14].

These secondary anti-inflammatory activities of macrolides, which are summarized in Table 2, appear to be particularly effective in controlling neutrophil-mediated inflammation. This, in turn, may explain the usefulness of these agents in the treatment of inflammatory conditions of both infective and non-infective origin such as chronic obstructive pulmonary disease, cystic fibrosis, diffuse panbronchiolitis, refractory asthma, and bronchiolitis obliterans syndrome, in which the neutrophil appears to be the major offender [42,43,44,45,46].

**Table 2:** Secondary anti-inflammatory activities of macrolides.

Target cell	Effect(s)
Neutrophils	↓ activity of NADPH-oxidase and production of reactive oxygen species. ↓ migratory activity due especially to inhibition of the production of IL-8 by other inflammatory cells and structural cells, as well as down-regulation of neutrophil adhesion molecules and their counter-receptors on vascular endothelium. Induction of apoptosis.
Monocytes/macrophages	↓synthesis of type II nitric oxide synthase. ↑clearance of apoptotic neutrophils.
	
Structural cells (airway epithelium, fibroblasts)	↓synthesis of IL-8 and TNF.

### 5.2. Anti-inflammatory activities of azole anti-mycotics

The azole class of anti-mycotic agents is widely used in clinical practice and has proven to be both safe and effective. Although primarily prescribed for their anti-fungal actions, these agents may also exhibit secondary anti-inflammatory properties by interacting with cells, such as neutrophils, which mediate the innate immune response. As mentioned above, activated neutrophils release toxic oxidants, proteolytic enzymes and lipid mediators which may damage surrounding tissues [47].

Human neutrophils activated by Ca^2+^-mobilizing stimuli such as PAF, generate significant quantities of arachidonic acid (AA) following cytosolic phospholipase A_2_ (cPLA_2_)-mediated mobilization of membrane phospholipids [48]. Importantly, AA serves as a substrate for 5-lipoxygenase (5-LO) which in turn generates leukotriene B_4_ (LTB_4_). The roles of PAF and LTB_4_ in promoting inflammation and tissue injury are well established, and these lipid mediators may contribute to the pathogenesis of diverse conditions such as asthma and sepsis [49]. Despite its important role during inflammation, the identification of agents which inhibit release of LTB_4_ by activated neutrophils has remained elusive. Recent evidence, however, indicates that 5-LO may represent an attractive target in activated neutrophils as this enzyme is highly sensitive to the azole anti-mycotics [50]. Azole-mediated inhibition of 5-LO confers anti-inflammatory properties on these agents by interfering with LTB_4_ generation. Attenuation of LTB_4_ release, in turn, hinders leucocyte recruitment to foci of inflammation and modulates metabolism of calcium (Ca^2+^), an important intracellular second messenger in these cells [51]. LTB_4_ produced inside neutrophils is released to the cell exterior where it sustains cytosolic Ca^2+^ transients by triggering activation of G protein-coupled receptors linked to phospholipase C. Activation of phospholipase C generates inositol triphosphate (IP_3_), a potent mobilizer of intracellular Ca^2+^. Interference with this autocrine amplification of IP_3_ and Ca^2+^ release by azoles down-regulates numerous pro-inflammatory Ca^2+^-dependent responses of activated neutrophils, including production of reactive oxygen species [52] and degranulation [53]. Furthermore, the release of matrix metalloproteinases (MMPs) by activated neutrophils is also a Ca^2+^-dependent process and therefore sensitive to modulation by agents which facilitate rapid clearance of Ca^2+^ from the cell cytosol [54]. 

Azole antimycotics exert their anti-inflammatory actions by inhibiting 5-LO. Although this is a class effect, the relative potency of different azoles is dependent on their ability to cross cell membranes and interact with an intracellular target. Structural differences in the azole molecules result in variable degrees of penetration and are probably responsible for some agents (itraconazole and posaconazole) displaying greater anti-inflammatory potential than others (ketoconazole and fluconazole) [55].

An interesting question that arises and which warrants further research is whether the combination of a macrolide and an azole may exert synergistic effects in down-regulating excessive inflammation. Critically-ill patients sometimes receive both a macrolide and an azole to combat the polymicrobial infections commonly occurring in this setting. However, as discussed above, the timing of administration of these agents may need to be synchronized with the patient’s immune response so that the phase of excessive inflammation is targeted. Some patients with sepsis proceed to a phase of marked immunosuppression associated with an increased risk of infection, including fungal infections [56]. Biomarkers such as IL-10, the soluble Triggering Receptor Expressed on Myeloid Cells (s-TREM) and HLA-DR expression by monocytes may facilitate prediction of those patients with sepsis who are likely to develop immuno-paresis [56]. Azole anti-mycotics may also benefit this subgroup of sepsis patients.

### 5.3. Anti-inflammatory properties of fluoroquinolones

Fluoroquinolones, particularly those with a cyclopropyl moiety at the N1 position of the quinolone core structure, such as ciprofloxacin, moxifloxacin, grepafloxacin, and sparfloxacin, have been reported to possess secondary anti-inflammatory properties, targeting the production of pro-inflammatory cytokines, both *in vitro* and *in vivo* [57,58]. Exposure of epithelial cells and cells of the innate immune system, especially monocytes/macrophages, to fluoroquinolones *in vitro*, usually at therapeutic and supra-therapeutic concentrations, is accompanied by decreased production of several pro-inflammatory cytokines such as IL-1, IL-6, IL-8 and TNF [57,58,59,60,61,62,63,64]. The therapeutic potential of these findings is supported by data, albeit limited, from animal models of experimental infection with microbial pathogens which were either susceptible or resistant to fluoroquinolones. In both cases, administration of fluoroquinolones was associated with decreased production of pro-inflammatory cytokines and improved survival [58,65]. Interestingly, these inhibitory effects of fluoroquinolones on the production of pro-inflammatory cytokines are associated with increased synthesis of the growth and colony-stimulating factors, IL-2, IL-3, and GM-CSF [58]. In a recently reported clinical study, Calbo and colleagues compared the circulating pro-inflammatory and anti-inflammatory profiles of patients with severe pneumococcal pneumonia treated with either ceftriaxone or levofloxacin [66]. They observed that relatively late in the course of therapy, TNF was selectively and significantly decreased in the circulation of levofloxacin-treated patients, although no differences were evident with respect to outcome between the two treatment groups [66].

Several molecular/biochemical mechanisms have been described which may account for the secondary, anti-inflammatory interactions of fluoroquinolones with immune and inflammatory cells, as well as with structural eukaryotic cells. These include: (i) elevation of intracellular 3'-5' cyclic adenosine monophosphate (cAMP) through inhibition of cAMP phosphodiesterase, and/or by increasing the expression of cyclooxygenase-2, with resultant increased production of prostaglandin E_2_ which in turn activates adenylyl cyclase via its interaction with EP receptors [67]; and (ii) interference with cytokine gene transcription consequent to inhibition of extracellular-regulated kinases 1/2, 46-k Da Janus kinase and nuclear factor-kappa B [68].

On a more cautious note, however, some fluoroquinolones, such as trovafloxacin and grepafloxacin, have been reported to possess pro-inflammatory properties, specifically augmentation of the chemotactic and pro-oxidative activities of neutrophils [69,70]. The pro-inflammatory activities of these fluoroquinolones, which may, at least in the case of trovafloxacin [69], predispose to tissue damage, appear to result from augmentation of intracellular signaling cascades involving p38 mitogen-activated protein kinase [70]. These augmentative effects on the pro-inflammatory activities of neutrophils are not, however, common to all fluoroquinolones. This contention is based on our own experience with moxifloxacin. We have observed that treatment of activated human neutrophils with moxifloxacin at concentrations of up to 10 μg/mL *in vitro* did not affect the production of reactive oxygen species, release of the primary granule protease elastase, or cytosolic calcium fluxes in these cells [71]. Others have reported similar findings with moxifloxacin [72], demonstrating that fluoroquinolones differ with respect to their immunomodulatory effects on neutrophils.

### 5.4. Anti-inflammatory properties of tetracyclines

The antibiotic and non-antibiotic anti-inflammatory properties of tetracyclines are well-recognized and underpin the therapeutic efficacy of these agents in the treatment of inflammatory skin disorders of both infective and non-infective origin. The non-antibiotic, anti-inflammatory actions of tetracyclines, especially doxycycline and minocycline, have been attributed to the inhibitory effects of these agents on neutrophil migration, by mechanisms which appear to target the tertiary granule protease, matrix metalloproteinase (MMP)-9 [73,74,75,76,77,78]. Although critically involved in trans-endothelial migration, excessive release of MMP-9, as may occur in acute, severe, bacterial infection, has been implicated in the pathogenesis of inflammation-mediated tissue damage and organ dysfunction including lung and brain injury in bacterial pneumonia and meningitis [74,79,80]. Tetracycline-mediated inhibition of MMP-9 appears to be achieved by several mechanisms including: (i) direct inhibition of MMP-9; (ii) inhibition of MMP gene expression; (iii) inhibition of neutrophil degranulation; and (iv) up-regulation of the synthesis of tissue inhibitors of MMPs [73,74,75,76,77,78]. These non-antibiotic actions of the tetracyclines are evident at therapeutically attainable concentrations of these agents, and are likely to contribute to the control of neutrophil-mediated, harmful, inflammatory responses in severe bacterial infection.

## 6. Key Future Issues

This review has hopefully created an awareness of the critical interactions, both beneficial and harmful, which may occur between antibiotics and the innate immune system of the infected host, and which are likely in some cases to affect the outcome of antimicrobial therapy. Although useful insights have originated from laboratory-based and experimental animal studies, many important issues remain unresolved. These need to be addressed to ensure that currently available antibiotics are utilized optimally. Some of the most pressing issues are as follows:

- Are antibiotics utilized optimally with respect to both selection of appropriate combinations of antimicrobial agents and timing of administration? For example, in severely ill patients, should an inhibitor of bacterial protein synthesis be used routinely in combination with a beta-lactam or other bacterial agent? If so, and circumstances permitting, should the protein synthesis inhibitor be given in advance of the bactericidal agent?
- Because of their impressive secondary anti-inflammatory properties, are macrolides the preferred class of protein synthesis inhibitors in this setting?
- When anti-fungal prophylaxis/therapy is necessary, should an imidazole anti-mycotic with a good safety profile be selected and used in combination with a macrolide/bactericidal agent to confer optimum antimicrobial/anti-inflammatory chemotherapy?
- While the use of corticosteroids as adjuncts to beta-lactams may be useful in the treatment of severe pneumococcal meningitis, could addition of a macrolide to this therapeutic regimen provide additional benefit, given the insensitivity of neutrophils to the anti-inflammatory actions of steroids [81].
- Can the measurement of circulating, host-derived markers of inflammation and infection such as IL-4, IL-6, IL-10, TGF-β, TNF/TNFR, CRP, SAA, procalcitonin (PCT), and sTREM, as well as HLA-DR expression on circulating monocytes, be used, in association with clinical scoring systems, to provide potentially valuable information regarding the pro-inflammatory status of the patient at the time of admission and during the course of antimicrobial therapy?
- A greater awareness of the therapeutic potential of antimicrobial peptides, such as cathelicidins and defensins, which possess significant immunomodulatory properties in addition to their antimicrobial activities. These peptides increase the production of chemokines and cytokines, and also activate tissue repair processes [82].

## 7. Conclusions

The primary consideration in the selection of an antibiotic is clearly the sensitivity of the target microbial pathogen. However, secondary considerations, especially the potential of the antibiotic to trigger/exacerbate harmful inflammatory responses may be of significance in certain clinical settings, particularly that of severe infection with a high bacterial load. In this setting, the benefits of seemingly appropriate antimicrobial chemotherapy may be countered by pro-inflammatory activity of the antibiotic. Based on laboratory, experimental animal, and limited clinical data, potential strategies to address this complex clinical problem include combining an inhibitor of bacterial protein synthesis, preferably one with secondary anti-inflammatory properties, *i.e.* a macrolide, with a cell-wall targeted agent. However, definitive proof of the efficacy of this and related strategies will require the demonstration of a favourable outcome from well-designed, multi-centre clinical trials. 
